# Neoadjuvant chemotherapy is associated with an altered metabolic profile and increased cancer stemness in patients with pancreatic ductal adenocarcinoma

**DOI:** 10.1002/1878-0261.13344

**Published:** 2022-12-05

**Authors:** Manoj Amrutkar, Caroline S. Verbeke, Anette Vefferstad Finstadsveen, Linda Dorg, Knut Jørgen Labori, Ivar P. Gladhaug

**Affiliations:** ^1^ Department of Pathology Oslo University Hospital Norway; ^2^ Department of Pharmacology, Institute of Clinical Medicine University of Oslo Norway; ^3^ Department of Pathology, Institute of Clinical Medicine University of Oslo Norway; ^4^ Department of Hepato‐Pancreato‐Biliary Surgery, Institute of Clinical Medicine University of Oslo Norway; ^5^ Department of Hepato‐Pancreato‐Biliary Surgery Oslo University Hospital Norway

**Keywords:** cancer stem cells, metabolism, neoadjuvant chemotherapy, pancreatic cancer, proteomics

## Abstract

The modest clinical benefits of neoadjuvant chemotherapy (NAT) in pancreatic ductal adenocarcinoma (PDAC) are associated with a lack of robust data on treatment‐induced changes in the tumor. To this end, comparative proteomic profiling of tumor tissue samples from treatment‐naïve (TN, *n* = 20) and NAT‐treated (*n* = 22) PDACs was performed. Differentially expressed proteins were identified and correlation with overall survival (OS) was performed. Tumors were also examined for histopathological changes and expression of cancer stem cell (CSC) markers. Serum from 33 matched patients was analyzed for metabolic markers. Cytotoxicity, proliferation, and expression of CSC markers were assessed in chemoresistant Panc‐1 and Mia PaCa‐2 cells. Of the 2265 proteins identified, 227 and 144 proteins showed significantly altered expression and differential phosphorylation, respectively, in NAT compared with TN samples. The majority of these were metabolism‐related proteins, and 14 of these correlated moderately with OS. NAT‐treated tumors and chemoresistant cancer cells showed increased expression of CSC markers. Serum ALDH1A1 was higher in NAT compared with TN. Differentially phosphorylated proteins were mainly involved in cytoskeleton organization, cell locomotion, motility, and migration, and 17 of these showed a strong positive correlation with OS. This study provides evidence of the effects of NAT on PDAC metabolism at both the tumor and the systemic levels. NAT‐treated tumors showed significantly lower expression of metabolic proteins, and patients who underwent NAT showed reduced serum lactate and high‐density lipoprotein‐cholesterol. Lastly, cancer cells that survived cytotoxic treatment expressed higher CSC markers, both *in vivo* and *in vitro*.

## Graphical abstract

The impact of neoadjuvant chemotherapy on human pancreatic ductal adenocarcinoma (PDAC) was investigated using comparative total‐ and phospho‐proteome analysis of treatment‐naïve and neoadjuvantly treated (NAT) PDACs. Levels of markers of metabolism and cancer stem cells (CSCs) were measured in matched serum samples and the expression of CSC markers was investigated in PDAC cells that survived cytotoxic treatment. Our data indicate that NAT treatment induces metabolic changes in patient with PDAC both at tumor and systemic levels.

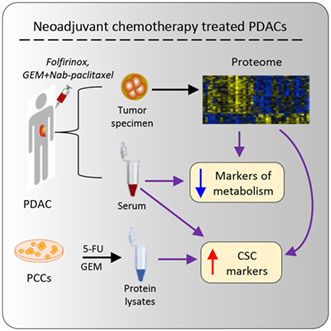

**Abbreviations**
5‐FU: 5‐fluorouracil
ALDH1A1: aldehyde dehydrogenase 1 family member A1
BRPC: borderline resectable pancreatic cancer
CA 19‐9: carbohydrate 19‐9 antigen
CAP: College of American Pathologists
CD44: cluster of differentiation 44
CSCs: cancer stem cells
DEPs: differentially expressed proteins
ECM: extracellular matrix
EpCAM: epithelial cell adhesion molecule
GEM: gemcitabine
GO: gene ontology
IMAC: immobilized metal affinity chromatography
LAPC: locally advanced pancreatic cancer
MS: mass spectrometry
NAT: neoadjuvant chemotherapy
OS: overall survival
PDAC: pancreatic ductal adenocarcinoma
PON1: paraoxonase 1
PRPC: primary resectable pancreatic cancer
TiO_2_
: titanium dioxide
TN: treatment‐naïve
TRG: tumor regression grading

## 1 Introduction

Over 90% of all pancreatic tumors are pancreatic ductal adenocarcinoma (PDAC), which is one of the deadliest solid tumor types [1]. Despite improvements in diagnosis and treatment strategies, in contrast to the improvement in prognosis that has been achieved for many solid cancers, the survival of PDAC patients remains extremely poor with 5‐year overall survival (OS) rate below 10% [2]. PDAC is expected to become the second leading cause of cancer‐related deaths in Western countries by the end of the present decade [2, 3, 4]. The only potentially curative treatment for PDAC is surgical resection followed by adjuvant chemotherapy; however, only 10–15% patients present with a localized resectable tumor [5]. The remaining patients (> 80%) are either inoperable because of metastatic disease (50–55%) or have potentially resectable tumors categorized as borderline resectable (BR) or locally advanced (LA) for which resectability largely depends on the response to preoperative, that is, neoadjuvant chemotherapy. In recent years, a multimodal approach to PDAC consisting of a sequence of neoadjuvant chemotherapy followed by surgery and adjuvant chemotherapy has been considered a possible treatment option for all primary resectable (PR), BR, and LA tumors [6]. The importance of neoadjuvant treatment (NAT) in PDAC is also underscored by the fact that only approximately 50% of patients are able to receive adjuvant chemotherapy, mostly due to surgical complications, disease progression, or poor performance status [7].

The preferred chemotherapeutic regimens for PDAC patients in both neoadjuvant and adjuvant settings include FOLFIRINOX (or modified FOLFIRINOX) and gemcitabine (GEM) with or without nab‐paclitaxel (Abraxane) [8, 9]. The desired effects of NAT include reduction of cancer burden, potential control of metastasis, and a generally safer selection of patients eligible for surgical resection [10, 11, 12, 13, 14, 15, 16, 17]. Despite these advantages, the role of NAT in PDAC remains debatable owing to the sparsity of robust data on its actual clinical benefits [9, 18, 19]. Moreover, the rapid development of resistance to chemotherapeutic agents, a hallmark of PDAC, remains a potential cause of concern when using NAT [20]. Therefore, a detailed understanding of the effects of NAT at the molecular and tissue level is crucial for further improvement of PDAC treatment strategies.

Several studies have reported the effects of NAT on histological features in surgical specimens with PDAC [21]. For routine clinical purposes, the effect of NAT is evaluated histologically by tumor regression grading (TRG), which is known to suffer from inter‐observer variation and poor correlation with patient outcome [22, 23, 24]. However, relatively little is known about the molecular changes induced by NAT and their potential association with the outcome. Since the availability of pre‐treatment tumor tissue is generally limited to exceedingly small samples from diagnostic procedures, the actual assessment of NAT‐induced effects on tumor tissue is generally restricted to comparisons between treatment‐naïve (TN) and NAT‐treated surgical resection specimens with PDAC. To this end, the investigation of treatment‐induced molecular changes using whole‐tissue proteome analysis seems an obvious approach. Proteomics has recently been employed for detailed identification and comparative analysis of protein expression in different tissue compartments and disease models, as well as for the identification of potential biomarkers in PDAC [25, 26, 27, 28]. This study investigated total‐ and phospho‐proteome of snap‐frozen tumor tissue samples obtained from surgical specimens of PDAC in patients who underwent surgery either up‐front (TN group) or following NAT. The aim of this study was to detect treatment‐induced changes in the tumor and to identify differentially expressed proteins (DEPs) and altered signaling pathways between TN and NAT tumors, and their potential correlation with different clinical parameters.

## 2 Materials and methods

### 2.1 Patients and samples

The study series consists of PDAC tissues sampled from surgical specimens of 42 patients who underwent resection at Oslo University Hospital (Rikshospitalet), Norway, between 2016 and 2018. Of these patients, 20 underwent upfront surgery (TN group), and 22 were treated with neoadjuvant chemotherapy prior to surgical resection (NAT group). Clinicopathological information on the study series is provided in Table 1 and Table S1. Survival data were last updated in December 2021. All procedures involving human participants were performed in accordance with the ethical standards of the institutional and/or national research committee and the Helsinki Declaration and its later amendments or comparable ethical standards. This study was approved by the Regional Ethics Committee of South‐Eastern Norway (REC South East, project number 2015/738). Written informed consent to use biomaterials and clinical information for research purposes (analysis and publication) was obtained from all patients included in this study.

**Table 1:** Clinicopathological features of the study series. Age, BMI, tumor size, serum parameters, and overall survival data are presented as median values and ranges, whereas the data for all other categories are presented as actual numbers and percentages. All patients had metastasis grade M0 at surgery. BMI, body‐mass index; CA 19‐9, carbohydrate 19‐9 antigen; CAP, College of American Pathologists; DP, distal pancreatectomy; PPPD, pylorus‐preserving pancreatoduodenectomy; TNM, tumor‐node‐metastasis; TP, total pancreatectomy.

Category	Treatment‐naïve (TN, *n* = 20)	Neoadjuvantly treated (NAT, *n* = 22)
Gender		
Men	7 (35%)	15 (68%)
Women	13 (65%)	7 (32%)
Age (years)	71.0 (52.0–80.0)	64.0 (53.0–73.0)*
BMI (kg/m^2^)	23.6 (16.6–39.6)	24.1 (16.9–34.8)
Comorbidity	16/20 (80%)	11/22 (50%)
Diabetes	7	3
Cardiovascular	7	2
Hypertension	8	5
Others	5	5
Disease stage at resection		
Primary resectable (PRPC)	18 (90%)	6 (27%)
Borderline resectable (BRPC)	2 (10%)	10 (46%)
Locally advanced (LAPC)	0	6 (27%)
Resection type		
PPPD	19 (95%)	14 (64%)
TP	0	7 (32%)
DP	1 (5%)	1 (4%)
Tumor size (mm)	36.0 (24.0–60.0)	35.5 (22.0–65.0)
TNM classification (8th edition)		
Tumor (T)		
T1	0	0
T2	17 (85%)	15 (68%)
T3	3 (15%)	7 (32%)
T4	0	0
Lymph node metastasis (*N*)		
N0	3 (15%)	2 (9%)
N1	8 (40%)	9 (41%)
N2	9 (45%)	11 (50%)
Tumor regression grade		
CAP‐0	–	0
CAP‐1	–	1 (4%)
CAP‐2	–	12 (55%)
CAP‐3	–	9 (41%)
Serum parameters		
CA 19‐9 preoperative (U·mL^−1^)	240.5 (5.0–11371.0)	55.5 (7.0–2380.0)^#^
Bilirubin (μmol·L^−1^)	27.0 (4.0–228.0)	6.0 (3.0–24.0)**
Albumin (mg·dL^−1^)	41.0 (35.0–45.0)	42.0 (34.0–44.0)
C‐reactive protein (mg·L^−1^)	2.9 (0.8–24.0)	2.3 (0.8–17.0)
Adjuvant chemo (postoperative)	11/20 (55%)	18/22 (82%)
Overall survival (months)	31.2 (4.1–56.1)	13.8 (1.5–38.9)**

*P < 0.05, **P < 0.01 and ^#^ *P* < 0.1 when compared between TN and NAT.

### 2.2 Proteomic analysis

**Fig. 1:**
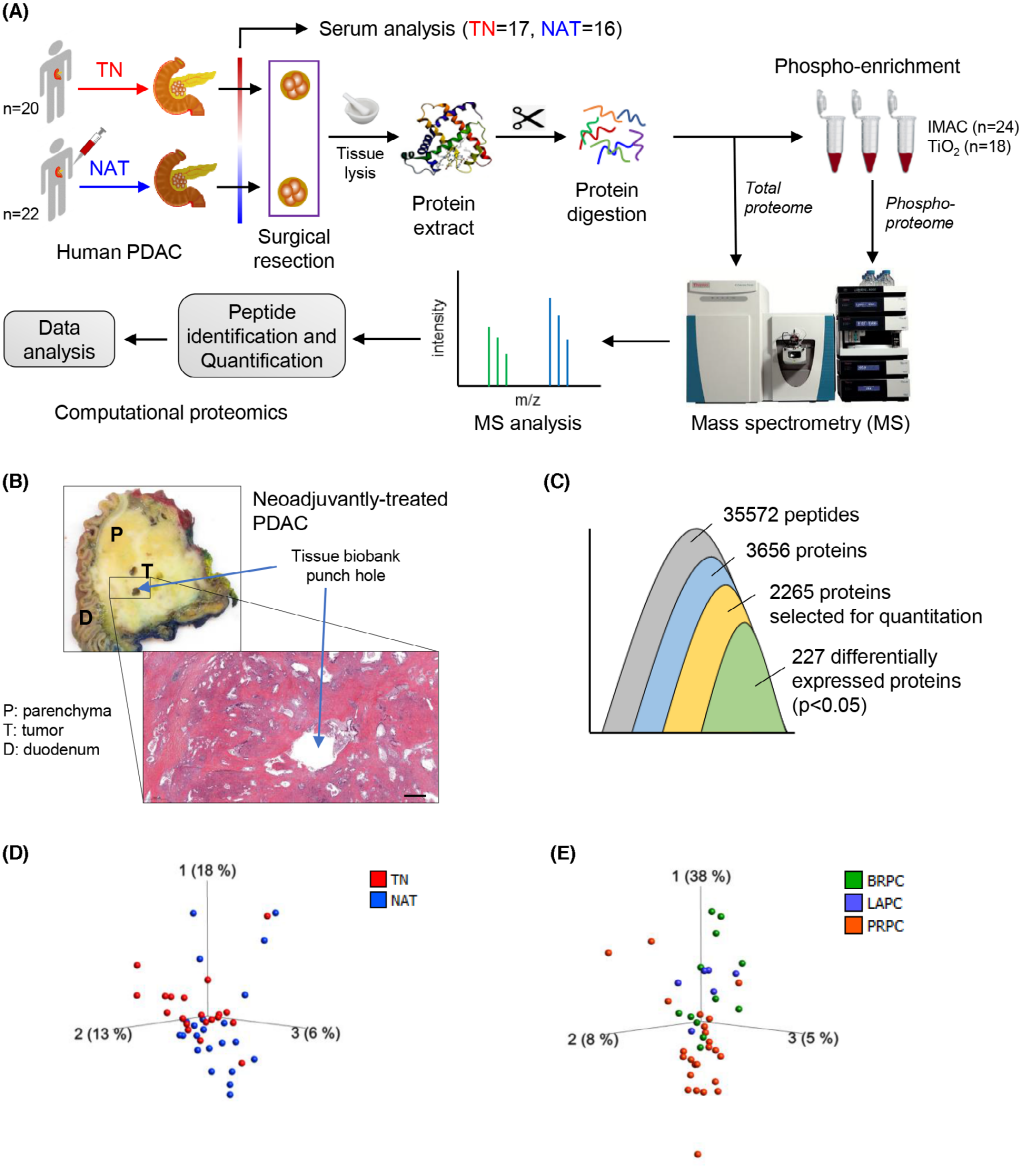
Study design and overview of the tissue proteome. (A) Schematic overview of the study procedure. (B) Macroscopic appearance of the surgical specimen with PDAC and representative picture of a hematoxylin and eosin‐stained tissue sample. Scale bar = 1000 μm. (C) Overview of the number of proteins that were identified, quantified, and processed. (D, E) PCA plots for sample distribution. Each dot represents an individual sample, which is colored according to the treatment group (D) and disease state (E). TN, treatment‐naïve; NAT, neoadjuvantly treated; PDAC, pancreatic ductal adenocarcinoma; IMAC immobilized metal affinity chromatography; TiO_2_, titanium dioxide; BRPC, borderline resectable pancreatic cancer (PC); LAPC, locally advanced PC; PRPC, primary resectable PC.

Tumor tissue extracts were subjected to mass spectrometry (MS) to identify total proteome and phosphorylated proteins. An overview of the experimental procedure is shown in Fig. 1A. Sample handling, protein digestion, phosphopeptide enrichment, MS and protein identification, quantitation, and data analysis were performed as described below.

#### 2.2.1 Sample handling

Immediately following surgical removal, the resection specimens were transported on ice from the operation room to the laboratory. An experienced pathologist identified the tumor bed and collected a 2‐mm^2^ tissue sample from each tumor within ~ 20 min after the surgical resection. Each tissue sample was placed in a pre‐labeled cryotube and was snap‐frozen in liquid nitrogen, following which all samples were stored at −80 °C until further use. For tissue homogenization, samples were crushed to powder form in a porcelain dish that was kept on dry‐ice and subsequently added to an Eppendorf tube with 1 mL cold urea lysis buffer containing 8 m urea, 75 mm sodium chloride, 50 mm Tris (pH 8.2), protease, and phosphatase inhibitors. Tissue homogenates were stored at −80 °C until further processing.

#### 2.2.2 Protein digestion and phosphopeptide enrichment

From each tissue homogenate, 2 mg of protein was used for protein digestion and for the subsequent enrichment of phosphopeptides. Samples were reduced with 5 mm dithiothreitol for 45 min at 37 °C and alkylated with 10 mm Iodoacetamide in the dark for 30 min at room temperature (RT) before digestion with 5 μg Lys‐C (Wako Chemicals, Richmond, VA, USA) for 2 h at RT. Samples were then diluted with 50 mm ammonium bicarbonate (NH_4_HCO_3_) to a final urea concentration of 1 m before digestion with trypsin (10 μg) over night for 37 °C. Of the resulting peptides, 70 μg was saved for full‐proteome analysis, while the remainder was used for phosphopeptide enrichment. Samples were enriched using either immobilized metal affinity chromatography (IMAC; *n* = 12 each of TN and NAT) or titanium dioxide (TiO_2_; *n* = 8 of TN and *n* = 10 of NAT). Samples for phospho‐proteomics were desalted on 50 mg Sep‐Pak Vac RP C18 cartridges (Waters, Milford, MA, USA). Phosphopeptides were enriched with PHOS‐Select Iron Affinity Gel (Sigma Aldrich, St. Louis, MO, USA) according to the manufacturer's instructions following the protocol described in Soderholm et al. [29] or using TiO_2_ beads as described in Casado et al. [30].

#### 2.2.3 Mass spectrometry analysis

The full‐proteome samples and enriched phosphopeptides were analyzed using an Easy nLC1000 nano‐LC system connected to a quadrupole—Orbitrap (QExactive Plus; Thermo Fisher Scientific, Waltham, MA, USA) mass spectrometer (ThermoElectron, Bremen, Germany) equipped with a nano‐electrospray ion source (EasySpray; Thermo Fisher Scientific). Peptides were separated on a EasySpray column (C18, 2 μm beads, 100 Å, 75 μm inner diameter; Thermo Fisher Scientific) capillary of 50 cm bed length using a 120‐min gradient up to 35% solvent B. Solvent A was aqueous 0.1% formic acid, whereas solvent B was 100% acetonitrile in 0.1% formic acid. The flow rate was 0.3 μL·min^−1^. Column temperature was kept at 60 °C.

The mass spectrometer was operated in the data‐dependent mode to automatically switch between MS and MS/MS acquisition. Survey full scan MS spectra (from *m/z* 300 to 1500) were acquired in the Orbitrap with resolution of 70 000 at *m/z* 200 (after accumulation to a target of 3 000 000 ions in the quadruple). This method allowed sequential isolation of the most intense multiply‐charged ions, up to 10, depending on signal intensity, for fragmentation on high‐energy collision dissociation (HCD) at a target value of 100 000 charges or maximum acquisition time of 100 ms. MS/MS scans were collected at 17500 resolution at the Orbitrap cell. Target ions already selected for MS/MS were dynamically excluded for 30 s. General MS conditions were as follows: electrospray voltage 2.1 kV; no sheath and auxiliary gas flow, heated capillary temperature of 250 °C, normalized HCD energy 25%.

#### 2.2.4 Protein and phosphorylation site identification, label‐free quantitation

Mass spectrometry raw files were submitted to maxquant software (RRID:SCR_014485) version 1.6.2.1 for protein identification, label‐free quantitation (LFQ) and phosphorylation site identification [31]. Parameters were set as following: carbamidomethyl (C) as fixed modification, protein *N*‐acetylation, phospho (STY) and methionine oxidation as variable modifications, first search error window of 20 p.p.m. and main search error window of 6 p.p.m. Trypsin without proline restriction enzyme option was used with two miscleavages allowed. Minimal unique peptides were set to 1, and false discovery rate (FDR) allowed was 0.01 (1%) for peptide and protein identification. The UniProt human database was used for protein identification. Generation of reversed sequences was selected to assign FDR rates.

The proteome and phospho‐proteome data were further processed using perseus version 1.6.1.3 (Max‐Planck Institute of Biochemistry, Martinsried, Germany). For LFQ, normalized intensities were log_10_ transformed, minimum 50% valid values in at least one group was required, missing values were imputed from normal distribution with default settings, and *t*‐test was done using permutation based FDR < 0.05 as the criteria. For the phospho‐proteome, the peptide intensity values from MaxQuant Phospho(STY)‐file were log_2_ transformed, minimum 50% values in at least one group was required, missing values were imputed from normal distribution with default settings, and *t*‐test was done considering *P* < 0.05 a statistically significant.

qlucore omics explorer version 3.7 (Qlucore AB, Lund, Sweden) was used for data exploration and visualization, principle component analysis (PCA), differential profiling (Heatmap) of the proteome, correlation between Kaplan–Meier survival estimates and protein expression [32, 33]. In addition, the list of DEPs were subjected to Kyoto Encyclopedia of Genes and Genomes (KEGG) database for pathway analysis [34], while Gene Ontology (GO) analysis was conducted using the DAVID (RRID:SCR_001881) Bioinformatics Database version 6.8 [35, 36].

### 2.3 Histopathological assessment

After securing tissue samples for proteomic analysis, the surgical specimen was fixed in 10% neutral‐buffered formalin for 48 h and examined by an experienced pancreatic pathologist according to international guidelines [37]. TRG was determined for PDACs treated with NAT, according to the College of American Pathologists (CAP) guidelines. CAP‐0 (complete response): no remaining viable cancer cells; CAP‐1 (moderate response): only small clusters or single cancer cells remaining; CAP‐2 (minimal response): residual cancer remaining, but with predominant fibrosis; CAP‐3 (poor response): minimal or no tumor kill with extensive residual cancer [38]. The tissue block representing the tumor bed area from which samples were taken for proteomics was used for immunohistochemical evaluation of cancer stem cell (CSC) marker expression. For the histochemical analysis of CSC markers, 10 representative tumor samples from each treatment group were selected based on their expression level observed in the tissue proteome and respective serum samples. For immunostaining, incubation with primary antibodies against ALDH1A1 (ab52492; RRID:AB_867566), CD44 (ab51037; RRID:AB_868936), and EpCAM (ab223582; RRID:AB_2762366) from Abcam, Cambridge, UK was followed by incubation with Immpress HRP anti‐Rabbit secondary antibodies (MP‐7401; RRID:AB_2336529) from Vector Labs (Newark, CA, USA) as previously described [39].

### 2.4 Serum analysis

Serum extracted from blood samples collected before surgery from PDAC patients in both TN (*n* = 17) and NAT (*n* = 16) groups were investigated for lactate (MET‐5012; Cell Biolabs, San Diego, CA, USA), pyruvate (MET‐5125; Cell Biolabs), cholesterol (total, high‐density lipoprotein [HDL] and low‐density lipoprotein [LDL]; ab65390; Abcam), ALDH1A1 (EH16RB; Invitrogen, Waltham, MA, USA), and PON1 (EH376RB; Invitrogen). All analyses were performed according to the manufacturer's instructions.

### 2.5 *In vitro* assessments

Human pancreatic cancer cell lines Panc‐1, Mia PaCa‐2, BxPC‐3, and HPAF‐II were purchased from ATCC (Manassas, VA, USA). All cell lines were maintained at 37 °C with 5% CO_2_ in a normal growth medium, that is, Dulbecco's modified Eagle's medium (DMEM) containing 4.5 g·L^−1^ d‐glucose (GlutaMAX™, #31966047), supplemented with 10% FBS (#10500064), and 1% each of penicillin–streptomycin (#15140122) and amphotericin B (#15290026). Media and supplements were purchased from Thermo Fisher Scientific. Cell cultures were routinely checked for mycoplasma using MycoAlert™ Mycoplasma Detection Kit (#LT07‐703; Lonza, Basel, Switzerland).

For experimental purpose, cells were seeded at a density of 5000 cells per well in 96‐well plates. Initially, dose response curves for both 5‐fluorouracil (FU) or GEM was determined by assessing MTT‐based cell viability in cells treated with varying concentrations of both drugs for 48 h. Next, cells were treated with both drugs at a single concentration of 10 μm for three repeated doses over a period of 8 days (see Fig. 6A for experiment timeline). During this period, cells were cultured and maintained in low glucose (1 g·L^−1^) DMEM GlutaMAX (#21885025; Thermo Fisher Scientific) containing 1% FBS. Drug‐induced cytotoxicity was measured using MTT viability assay, and the anti‐proliferative response was evaluated using BrdU incorporation assay. Both MTT and BrdU reagent were added into respective wells 4 h prior to the endpoint. For MTT assay, formation of purple formazan crystals by metabolically active cells was determined using spectrophotometry, as described previously [40]. The incorporation of BrdU into actively proliferating cells was measured using BrdU Cell Proliferation ELISA Kit (ab126556; Abcam).

### 2.6 Western blot analysis

Protein expression was measured using western blot analysis, as described previously [41]. Briefly, proteins were separated by electrophoresis (SDS/PAGE) and transferred using a semi‐dry transfer system (Bio‐Rad, Hercules, CA, USA). This was followed by overnight incubation with listed primary antibodies at 4 °C, and subsequently with secondary antibodies for 1 h at RT. Primary antibodies against ALDH1A1 (ab52492; RRID:AB_867566), CD44 (ab51037; RRID:AB_868936), EpCAM (ab223582; RRID:AB_2762366), and PON1 (ab92466; RRID:AB_10562283) were purchased from Abcam while Vinculin (#13901; RRID:AB_2728768) and GAPDH (#5174; RRID:AB_10622025) from Cell Signaling Technology (Danvers, MA, USA). HRP‐conjugated Goat anti‐Rabbit secondary antibody from Bio‐Rad (1706515; RRID:AB_11125142) was used.

### 2.7 Statistical analysis

Proteome data were analyzed using qlucore omics explorer version 3.7 and DAVID Bioinformatics platform version 6.8. Correlations between protein expression and survival or other clinical parameters were assessed using Spearman's correlation test. Results from biochemical assessments and *in vitro* experiments are expressed as mean ± SEM. Statistical analysis of these results was performed using graphpad prism version 6 (GraphPad Software, San Diego, CA, USA) (RRID:SCR_002798), using an unpaired two‐tailed Student's *t*‐test, with *P* < 0.05 considered statistically significant.

## 3 Results

### 3.1 Clinical assessment of source tumors

Macroscopic appearance of the PDAC, location of sample collection, and corresponding histology image are shown in Fig. 1B. The detailed clinical features of the patients included in this study are provided in Table 1 and Table S1. In the TN group, 90% of patients had a primary resectable tumor, whereas the majority of patients (73%) in the NAT group had BR or LA tumors. Neoadjuvant chemotherapy regimens included FOLFIRINOX, alone (*n* = 15) or followed with GEM plus Abraxane (*n* = 4) or FLOX (*n* = 1), and two patients received either GEM alone (*n* = 1) or GEM plus Abraxane (*n* = 1; Table S1). Histopathological evaluation revealed a higher proportion of T3‐tumors in the NAT group (32%) than in the TN group (15%). Preoperative serum carbohydrate 19‐9 antigen (CA 19‐9) levels were almost 5‐fold lower in NAT (269 ± 114 U·mL^−1^) than in TN (1307 ± 590 U·mL^−1^), although this was not statistically significant (*P* = 0.08; Table S1). Serum bilirubin levels were also lower in NAT compared to TN (5.7‐fold, *P* < 0.01), whereas no differences in albumin and C‐reactive protein levels were observed between the groups (Table 1).

### 3.2 Total proteome explorative analysis

An overview of the proteins identified, quantified, and analyzed is shown in Fig. 1C. MS identified 35 572 peptides corresponding to 3656 proteins. For further quantitation, an individual protein was included only if it was detected in at least half of the samples from each group. By applying this criterion, 2265 proteins mapping to 31 221 peptides were selected. Enrichment analysis of these proteins revealed their involvement in a broad range of biological processes, including cell adhesion, metabolic regulation, extracellular matrix (ECM) organization, and cell proliferation. The complete MS data and a list of enriched biological processes and molecular functions are provided in Table S2. Data visualization was carried out using a 3D‐PCA plot for samples (Fig. 1D), which showed some overlap in the sample distribution between two groups and indicated heterogeneity between samples in each treatment group. Furthermore, analyzing the sample distribution according to the disease stage in terms of resectability (PRPC, BRPC, LAPC), irrespective of the treatment group, revealed no clear pattern (Fig. 1E). Of note, the majority of the samples from PRPC appeared to differ from the rest; however, a certain overlap with LAPC or BRPC was present, and the small sample size in each group limited the extent of possible differences.

### 3.3 Comparing total proteome between TN and NAT samples

**Fig. 2:**
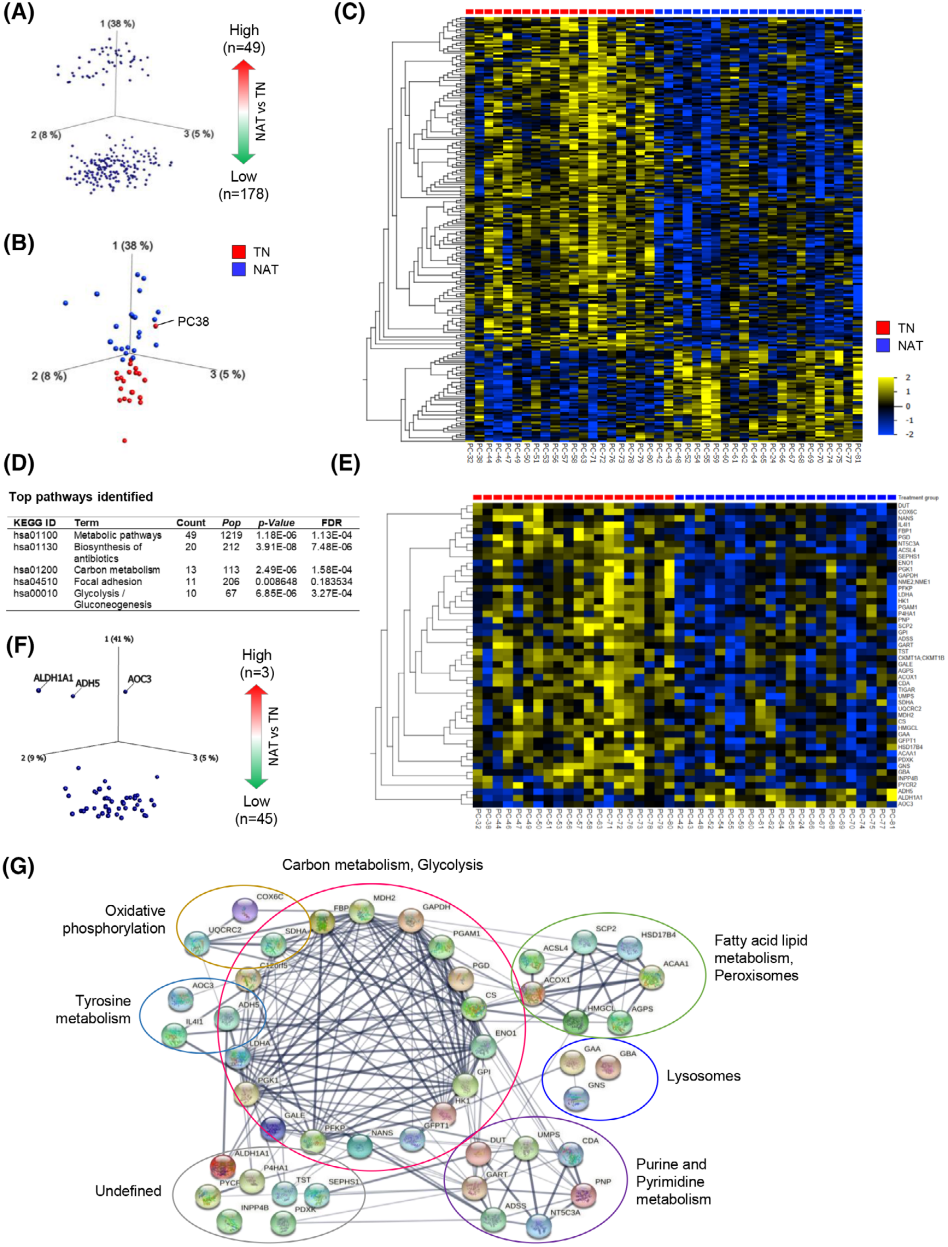
Comparison of the total proteome between treatment‐naïve (TN) and neoadjuvantly treated (NAT) pancreatic cancers. (A–C) Distribution of differentially expressed proteins in NAT and TN tumors. (A) PCA‐variable plot, each dot represents an individual protein. (B) PCA‐sample plot, each dot represents an individual sample, which is colored according to the treatment group. (C) Heatmap showing the distribution of all 227 proteins with differential expression in NAT and TN. (D) List of major KEGG pathways identified. (E, F) Differentially expressed metabolic pathway proteins shown as a heatmap (E) and as a PCA‐variable plot (F). In C and E, samples were distributed according to the treatment group, and variables (proteins) were arranged according to hierarchical clustering. (G) STRING network of differentially expressed metabolic pathway proteins.

Comparing the total proteome between NAT and TN samples revealed 227 DEPs, which accounted for ~ 10% of all proteins analyzed (Fig. 1C, Table S3). Next, the distribution of these proteins was visualized using a PCA‐variable plot, in which each variable corresponded to an individual protein (Fig. 2A). The PCA plot showed a clear pattern, with the expression of 49 and 178 proteins being significantly higher or lower, respectively, in the NAT than in the TN group. Furthermore, a PCA‐sample plot of DEPs confirmed a clear distribution of samples according to treatment group (Fig. 2B). A heatmap (Fig. 2C) shows the hierarchical clustering of DEPs and treatment‐based sample distribution. Next, pathway analysis of these proteins was performed using the DAVID bioinformatics tool. Interestingly, 49 of the 227 DEPs (~ 22%) belonged to a single KEGG pathway term: metabolic pathways. Other major pathway terms identified included biosynthesis of antibiotics, carbon metabolism, focal adhesion, and glycolysis/gluconeogenesis (Fig. 2D, Table S3). The expression pattern of the 49 proteins associated with metabolic pathways is shown in a heatmap (Fig. 2E) and a PCA‐variable plot (Fig. 2F). Interestingly, the expression of only three proteins was significantly higher in NAT than in TN, namely ADH5, ALDH1A1, and AOC3. All three are oxidoreductase enzymes. Functionally, ADH5 and ALDH1A1 contribute to lipid metabolism, whereas AOC3 is involved in cell adhesion. The remaining 46 metabolic proteins with a significantly lower expression in NAT compared with TN are involved in diverse biological processes, such as canonical glycolysis, gluconeogenesis, fatty acid (beta) oxidation, and the alpha‐linolenic acid metabolic process. These proteins were subsequently grouped according to the KEGG pathways to which they belonged and presented as a STRING network (Fig. 2G). The detailed functional annotation of metabolism‐related DEPs is provided in Table S3.

Next, the DEPs were investigated for expression patterns according to tumor resectability, irrespective of treatment status (Fig. S1). Of the 227 proteins, 57 showed significantly different expression in BRPC and LAPC compared with PRPC (*P* < 0.01), as shown in a heatmap (Fig. S1A). The top 10 of these proteins with the most divergent expression includes a significantly high expression of APOA1, APOA2, and AOC3, and a significantly lower expression of CTNND1, PUF60, SFXN3, SERPINB1, SERPINB5, STXBP2, and VDAC1 in both BRPC and LAPC compared with PRPC samples (Fig. S1B,C). Functionally, APOA1, APOA2, and VDAC1 contribute to cholesterol metabolism, whereas AOC3 and CTNND1 are involved in cell adhesion. PUF60 contributes to apoptosis and transcriptional regulation, SFXN3 is a mitochondrial serine transporter, SERPINB1 regulates the innate immune response, SERPINB5 is a tumor suppressor, and STXBP2 regulates exocytosis.

### 3.4 Correlation between protein expression and survival

**Fig. 3:**
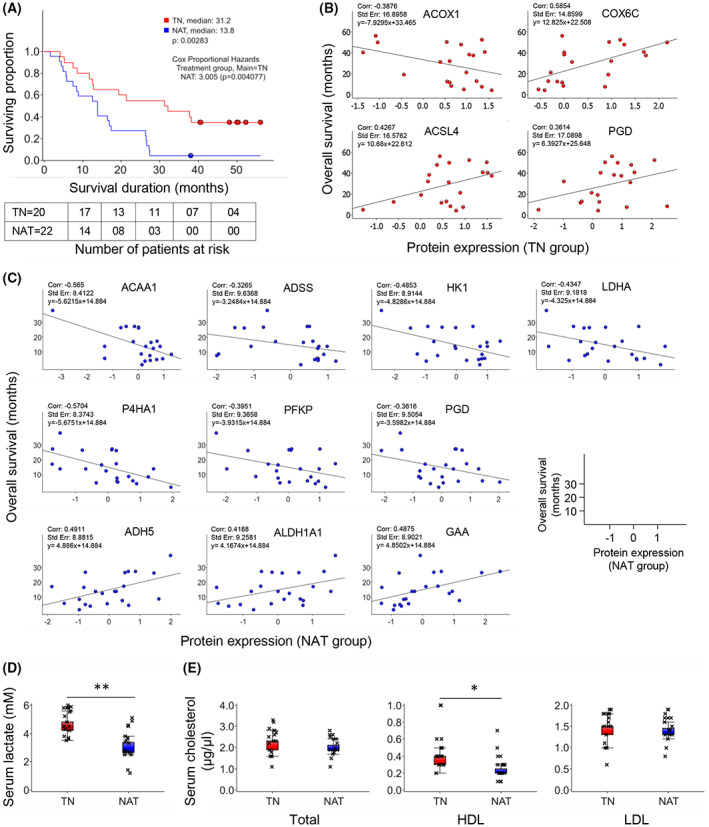
Survival analysis and correlation with protein expression and serum levels of metabolic markers. (A) Kaplan–Meier survival estimation. Survival duration was calculated from the date of surgery. (B, C) Scatterplots showing the correlation between the expression of individual metabolism‐related proteins (LFQ intensity) and overall survival for the TN group (B) and the NAT group (C). (D, E) Levels of lactate (D) and cholesterol (total, HDL and LDL, E) in serum samples obtained from patients in the TN (*n* = 17) and NAT (*n* = 16) group. For D and E: *P < 0.05 and **P < 0.01 comparing TN and NAT samples, *n* = 3, error bars indicate SEM, unpaired two‐tailed Student's *t*‐test. NAT, neoadjuvantly treated; TN, treatment‐naïve; HDL, high‐density lipoprotein; LDL, low‐density lipoprotein.

Patients undergoing NAT had a significantly lower post‐surgical OS compared to TN patients. The median OS for NAT and TN was 13.8 and 31.2 months, respectively (Fig. 3A). The number of patients surviving 2.5 years since the date of surgery differed significantly between two groups, with 1 in 22 and 11 in 20 for NAT and TN, respectively (Table S1). Of note, comparison of the median OS of all patients based on tumor resectability stage revealed the following ranking: PRPC > BRPC > LAPC (21.2 > 13.8 > 4.4 months; Fig. S1D). Potential correlations between metabolism‐related DEPs (identified in Fig. 2D,E) and OS were investigated separately for the TN (Fig. 3B) and NAT group (Fig. 3C). Of the 49 proteins investigated, only 4 and 10 proteins showed moderate correlation with OS in the TN and NAT group, respectively. The expression of ACSL4, COX6C and PGD correlated positively, while ACOX1 correlated negatively with OS (Fig. 3B). Functionally, ACOX1 and ACSL4 contribute to fatty acid lipid metabolism, whereas COX6C and PGD participate in oxidative phosphorylation and decarboxylation, respectively. In the NAT group, the expression of ADH5, ALDH1A1, and GAA correlated positively, whereas ACAA1, ADSS, HK1, LDHA, P4HA1, PFKP, and PGD expression correlated negatively with OS (Fig. 3C). These proteins are involved in diverse biological processes such as fatty acid lipid metabolism (ADH5, ALDH1A1, ACAA1), glycolysis (HK1, PFKP, LDHA), purine biosynthesis (ADSS), glycogen degradation (GAA), oxidative decarboxylation (PGD), and collagen biosynthesis (P4HA1). Besides metabolic proteins, other DEPs were also investigated for their potential correlation with OS. Of all 227 DEPs, the expression of 58 DEPs in the NAT group and 39 DEPs in the TN group showed moderate correlation (*r* > 0.3 or *r* < −0.3). An overview of all DEPs and their correlation with OS is provided in Fig. S2A. A list of proteins with *r* > 0.4 or *r* < −0.4, and their role in a variety of biological processes is presented in Table 2, and the respective correlation plots are provided in the Fig. S2B,C.

**Table 2:** List of differentially expressed proteins that correlate with overall survival. Information related to biological processes was obtained from the UniProt and GeneCards web platforms. Data are presented for proteins with correlation coefficient (*r*) values > 0.4 or < −0.4 in both the TN and NAT group.

Gene symbol	Protein name	*r* Value	Biological process
**Treatment‐naïve (TN)**			
COX6C	Cytochrome *c* oxidase subunit 6C	0.59	Oxidative phosphorylation
OLFML1, OLFML3	Olfactomedin‐like protein 1 and 3	0.48	Developmental proteins
FBLN1	Fibulin‐1	0.46	Extracellular matrix organization
ACSL4	Long‐chain‐fatty‐acid—CoA ligase 4	0.43	Fatty acid and lipid metabolism, beta oxidation
LAMC2	Laminin subunit gamma‐2	0.43	Cell adhesion, migration
A2M	Alpha‐2‐macroglobulin	0.41	Protease inhibitor
CAV1; CAV3	Caveolin‐1; Caveolin‐3	−0.42	Cell growth, migration, cholesterol distribution
SFN	14–3‐3 protein sigma	−0.43	Tumor suppressor, cell growth
LRRFIP1	Leucine‐rich repeat flightless‐interacting protein 1	−0.52	Transcriptional repressor. DNA binding
**Neoadjuvantly treated (NAT)**			
TM9SF3	Transmembrane 9 superfamily member 3	0.56	Beta‐adrenergic ligand binding, small molecule transporter
RBM47	RNA‐binding protein 47	0.52	RNA‐binding, p53‐p21 axis regulation
ADH5	Alcohol dehydrogenase class‐3	0.49	Lipid metabolism
GAA	Lysosomal alpha‐glucosidase	0.49	Lysosomal glycogen degradation
GSTM2	Glutathione S‐transferase Mu 2	0.47	Lipid metabolism
PPME1	Protein phosphatase methylesterase 1	0.47	Demethylation
CLU	Clusterin	0.45	Apoptosis, immune modulation, lipid transport
DDAH2	Dimethylarginine dimethylaminohydrolase 2	0.44	Nitric oxide synthase activity inhibitor
KTN1	Kinectin	0.43	Organelles and vesicles transporter
ALDH1A1	Aldehyde dehydrogenase 1A1	0.42	Lipid metabolism, oxidation, cancer cell stemness
GSTO1	Glutathione *S*‐transferase omega‐1	0.41	Cellular redox homeostasis
KIF5B	Kinesin‐1 heavy chain	0.41	Protein coding, nucleotide‐binding
STOML2	Stomatin‐like protein 2, mitochondrial	0.41	Mitochondrial biogenesis and ATP production
SYAP1	Synapse‐associated protein 1	0.41	Cell differentiation, cellular response to growth stimuli, TORC2 signaling
ANXA11	Annexin A11	−0.42	Cell cycle, calcium‐binding
ACTR3	Actin‐related protein 3	−0.43	Cell motility, ATP‐binding
LDHA	l‐lactate dehydrogenase A chain	−0.43	Glycolysis, pyruvate metabolism
SSBP1	Single‐stranded DNA‐binding protein, mitochondrial	−0.43	Mitochondrial biogenesis, DNA replication
EIF6	Eukaryotic translation initiation factor 6	−0.45	80S ribosome formation, cell growth, fatty acid biosynthesis, glycolysis
TAPBP	Tapasin	−0.45	Antigen processing and presentation MHC class I
EIF3D	Eukaryotic translation initiation factor 3 subunit D	−0.46	Translational initiation
HN1	Hematological and neurological expressed 1 protein	−0.46	Cell cycle and cell adhesion
RPS12	40S ribosomal protein S12	−0.46	Translational initiation
RPS21	40S ribosomal protein S21	−0.47	Translational initiation
S100A11	Protein S100‐A11	−0.47	Cell adhesion, motility, cell proliferation
HK1	Hexokinase‐1	−0.48	Glycolysis, inflammatory response, immunity
GCN1L1	Translational activator GCN1	−0.49	Stress response, translation regulation
VASP	Vasodilator‐stimulated phosphoprotein	−0.51	Cell adhesion, motility, integrin‐extracellular matrix interaction
COLGALT1	Procollagen galactosyltransferase 1	−0.52	Collagen fibril organization
CRIP1	Cysteine‐rich protein 1	−0.54	Cell proliferation, apoptosis, zinc absorption and transport
ACAA1	3‐Ketoacyl‐CoA thiolase, peroxisomal	−0.57	Fatty acids, alpha‐linolenic acid, and lipid metabolic process, beta oxidation
P4HA1	Prolyl 4‐hydroxylase subunit alpha‐1	−0.57	Collagen fibril organization, oxidation–reduction process
CORO1C	Coronin‐1C;Coronin	−0.58	Cell cycle, actin cytoskeleton organization, cell migration

### 3.5 Impact of NAT on systemic metabolism

To investigate the possible impact of chemotherapy on systemic metabolism, serum samples collected immediately prior to surgical resection of patients in both the TN (*n* = 17) and NAT (*n* = 16) group were analyzed for pyruvate, lactate, and cholesterol (total, HDL, and LDL). Serum lactate levels were significantly lower (1.5‐fold, *P* < 0.01) in the NAT group than in the TN group (Fig. 3D). However, pyruvate was below detectable levels in all samples. The serum levels of total‐ and LDL‐cholesterol were similar in TN and NAT samples, whereas serum HDL‐cholesterol was significantly lower (1.6‐fold, *P* < 0.05) in NAT than in TN samples (Fig. 3E).

### 3.6 Total proteome in stromal context

**Fig. 4:**
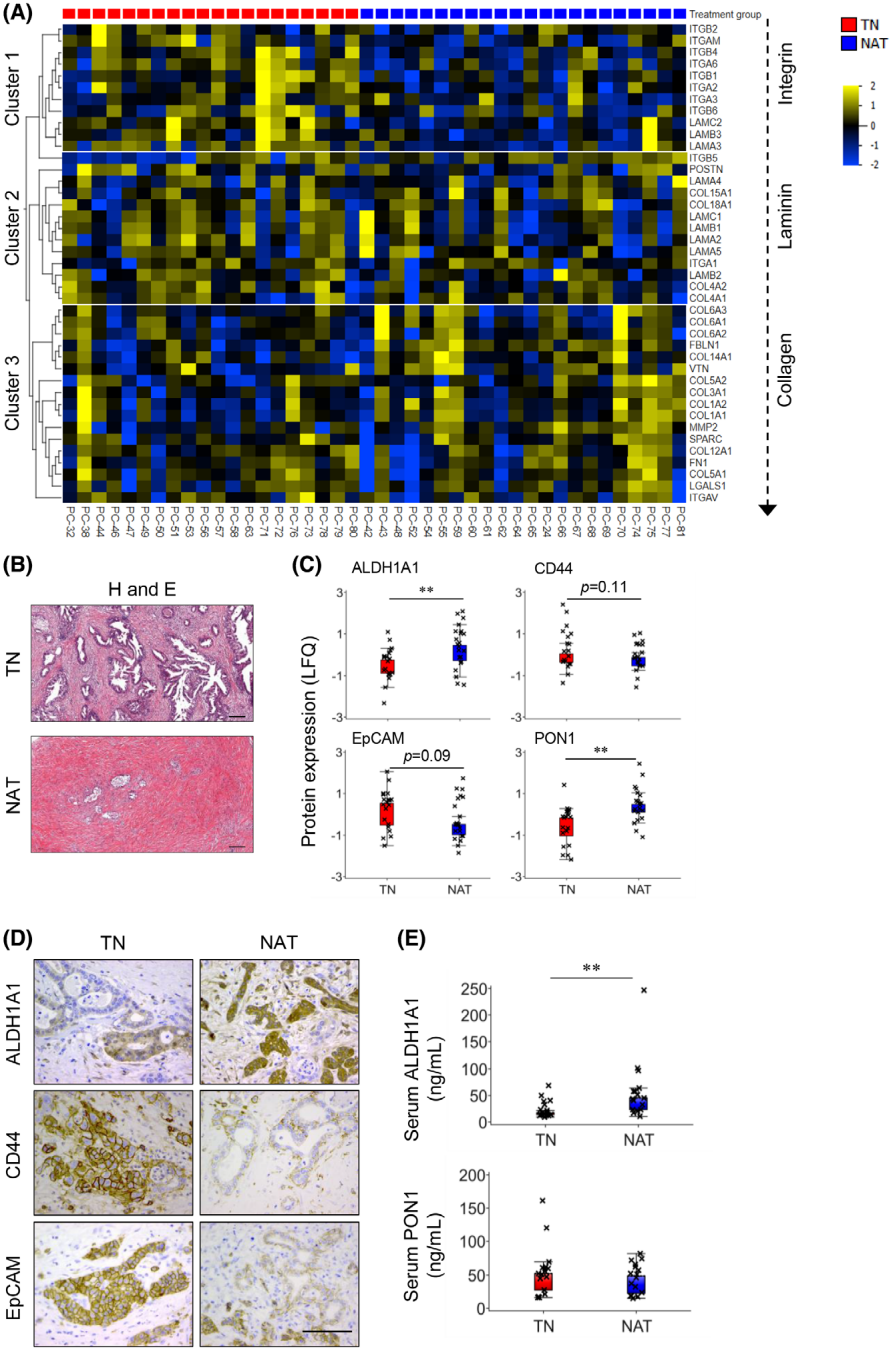
Proteome in stroma context; expression analysis of cancer stem cell (CSC) markers in tissue and serum. (A) Heatmap showing the expression pattern of extracellular matrix‐associated proteins. Samples and proteins arranged according to fold enrichment and arranged by treatment group and hierarchal clustering, respectively. (B) Representative pictures of hematoxylin and eosin (H and E)‐stained treatment‐naïve (TN) and neoadjuvantly treated (NAT) PDAC tissue samples. Scale bar = 200 μm. (C) Box plots showing the expression of CSC markers in TN (*n* = 20) versus NAT (*n* = 22) samples. (D) Representative pictures of immunohistochemical staining for ALDH1A1, CD44, and EpCAM. Scale bar = 100 μm. (E) Levels of ALDH1A1 and PON1 in serum samples obtained from patients in the TN (*n* = 17) and NAT (*n* = 16) groups. *n* = 3. For C and E, unpaired two‐tailed Student's *t*‐test with **P < 0.01 comparing between TN and NAT samples, error bars indicate SEM. ALDH1A1, aldehyde dehydrogenase 1 family member A1; CD44, cluster of differentiation 44; EpCAM, epithelial cell adhesion molecule; PON1, paraoxonase 1.

Next, the total proteome was investigated specifically for stromal and ECM‐related proteins. The expression profiles of these proteins are shown in the heatmap in Fig. 4A, which show a tentative pattern consisting of three clusters. Indeed, when considering the protein expression ratio of NAT to TN for each protein, a ranking integrins < laminins < collagens became apparent. A lower expression of various integrins and a higher expression of different collagens, fibronectin, vitronectin, fibulin‐1, SPARC, and MMP‐2 was observed in NAT compared to TN. Only five of these proteins were found to be included in the list of DEPs (Fig. 2C; *P* < 0.05): fibulin‐1 (FIBL‐1), integrin alpha‐6 (ITGA6) and beta‐4 (ITGB4), laminin subunit alpha‐3 (LAMA3), and gamma‐2 (LAMC2). Furthermore, the majority of PDACs in the NAT‐group were classified as CAP‐2 (55%) and CAP‐3 (41%; Table 1), indicating that tumor regression, the desired response to NAT, was either partial (CAP‐2) or poor/absent (CAP‐3). Representative images of H&E‐stained TN and NAT tissue samples shown in Fig. 4B illustrate the increase in fibrotic stroma that is associated with NAT‐induced tumor regression.

### 3.7 Impact of NAT on cancer stemness

Growing evidence suggests that PDAC after NAT may contain increased number of cancer cells with stem cell properties, whose persistent growth may result in a chemoresistant phenotype [42, 43]. Hence, the total proteome was explored for all known CSC markers, which revealed four markers: ALDH1A1, CD44, EpCAM, and paraoxonase 1 (PON1). The protein expression profiles of these CSC markers are shown in Fig. 4C. Interestingly, the expression of ALDH1A1 and PON1 was significantly higher in NAT than in TN (*P* < 0.01), suggesting a higher proportion of cancer cells with stem cell properties in the residual tumors following NAT compared with TN tumors. Immunohistochemical analysis confirmed the expression of ALDH1A1, CD44, and EpCAM in both TN and NAT tumor tissues (Fig. 4D). Furthermore, ALDH1A1 and PON1 levels were assessed in serum samples collected from patients in the TN (*n* = 17) and NAT (*n* = 16) group. A significantly higher level of serum ALDH1A1 (2.4‐fold, *P* < 0.05) was observed in the NAT than in the TN group (Fig. 4E). However, serum PON1 levels were similar in both groups (Fig. 4E).

### 3.8 Phospho‐proteome explorative analysis

**Fig. 5:**
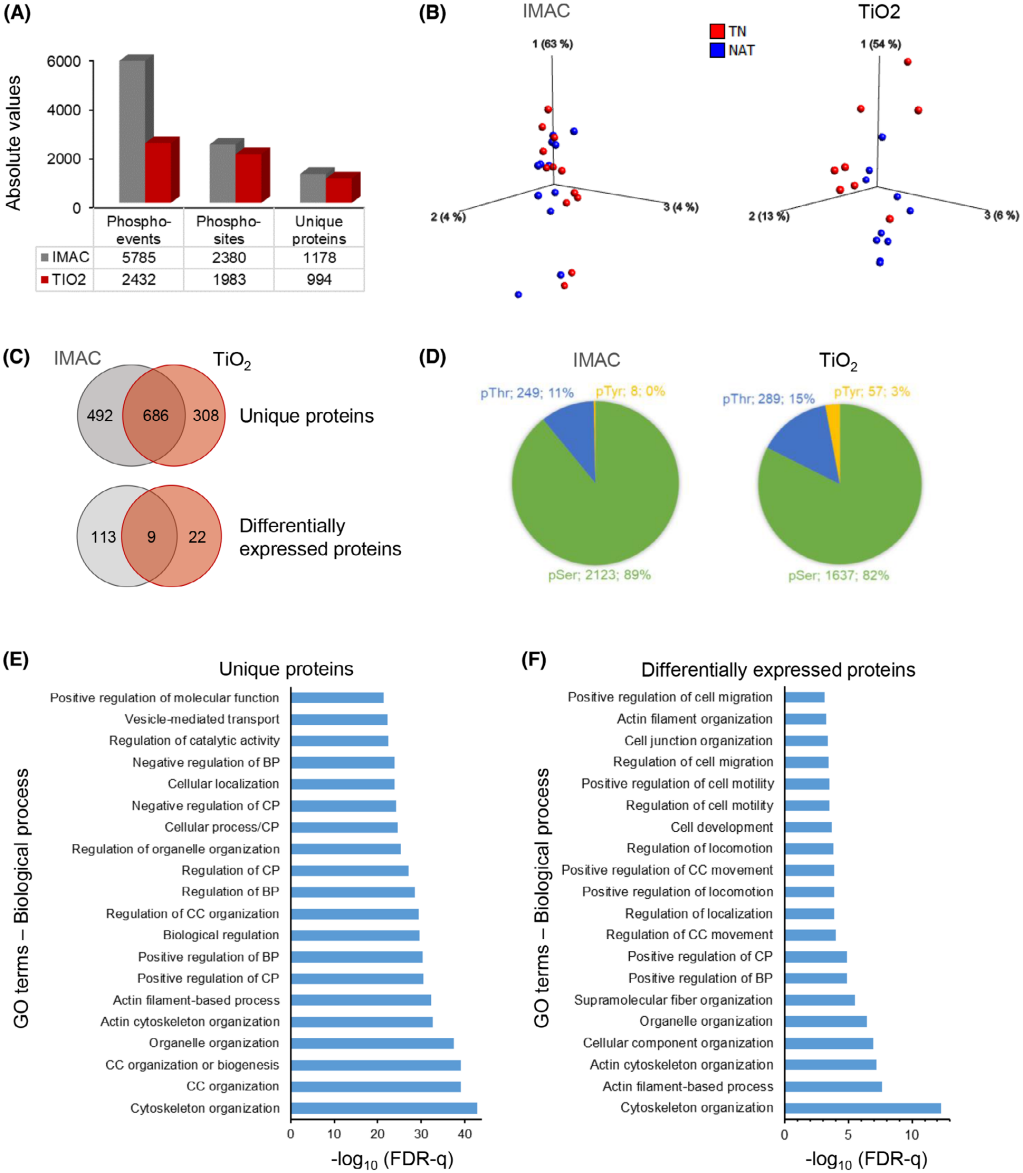
Analysis of the phospho‐proteome. (A) Overview of all phospho‐events, phospho‐sites, and unique proteins identified in PDAC tissue extracts enriched by IMAC (*n* = 12 each of TN and NAT) or TiO_2_ (TN = 8 and NAT = 10). (B) Individual PCA‐sample plot for IMAC and TiO_2_, samples colored according to the treatment group. (C) Venn diagram showing the distribution of phosphoproteins expressed in IMAC‐ or TiO_2_‐enriched samples. (D) Pie‐chart showing the distribution of phosphorylated amino acids, including the number and percentage of corresponding unique proteins. (E, F) Functional enrichment–gene ontology (GO) terms for biological processes for all phosphorylated proteins (E) and for proteins with altered phosphorylation in NAT compared to TN (F), irrespective of IMAC enrichment or TiO_2_ enrichment. IMAC, immobilized metal affinity chromatography; TiO_2_, titanium dioxide; NAT, Neoadjuvantly treated; TN, treatment‐naïve; BP, biological process; CC, cellular component; CP, cellular process; FDR, false discovery rate.

Immobilized metal affinity chromatography (*n* = 24) and TiO_2_ (*n* = 18) enriched phosphopeptide samples were analyzed using MS. An overview of phospho‐proteome identified is provided in Fig. 5A, and complete MS data are provided in Table S4. PCA sample plots were used to visualize the data from both enrichment procedures, which showed a considerable overlap among IMAC‐enriched samples, whereas TiO_2_‐enriched samples showed less overlap between the two groups. Moreover, both plots indicate a heterogeneous distribution of samples from each treatment group.

A total of 686 phosphorylated proteins were common to both enrichment methods, which accounted for 58% and 69% of all phosphorylated proteins from IMAC and TiO_2_, respectively (Fig. 5C). Analysis of the phospho‐proteomes from both treatment groups identified 171 phospho‐events from 122 proteins in IMAC and 41 phospho‐events from 31 proteins in TiO_2_ which were differentially expressed (Fig. 5C). Volcano plots visualizing the distribution of these proteins are presented in the Fig. S3A. For the majority of these phospho‐events (98% in IMAC and 87% in TiO_2_), phosphorylation was higher in NAT than in TN (Table S5). Only nine of the differentially expressed phosphoproteins: AKAP12, CALD1, CHGA, FLNA, IRF2BPL, MARCKS, NUCKS1, PGM5, and PPFIBP1 were common to both enrichment methods. The distribution of phospho‐acceptor residues revealed serine (pSer) as the predominant residue, representing 89% and 82% of all phospho‐events in IMAC and TiO_2_, respectively (Fig. 5D), followed by threonine (pThr; 11% and 15%) and tyrosine (pTyr; < 1% and 3%).

Next, the individual lists of unique proteins mapped to phosphopeptides identified in IMAC and TiO_2_ were merged and duplicate proteins were removed. To determine the association of these unique proteins with biological processes, enrichment analysis was performed. The most frequently enriched GO terms identified included organization of cytoskeleton, cellular components and organelles, as well as regulation of biological and cellular processes and molecular functions (Fig. 5E). Similarly, analysis for proteins with altered phosphorylation revealed enrichment of GO terms such as cytoskeleton organization, regulation of cell locomotion, motility and migration, and regulation of cellular and biological processes (Fig. 5F). Of note, only eight of the differentially expressed phosphoproteins are known to be associated with metabolic processes: lipid/cholesterol metabolism (ACLY, LIMA1, PITPNB), glucose metabolism (PGM5), glycolysis (ALDOA), purine metabolism (AMPD2), superoxide metabolism (SH3PXD2A), and insulin‐stimulated glucose transport (SORBS1).

### 3.9 Correlation between phosphoprotein expression and survival

An overview of the phosphoproteins that were differentially expressed in TN and NAT samples and that correlate with OS is provided in Fig. S3B. As shown in Table 3, in the NAT group, a strong positive correlation (*r* > 0.7) was observed for three phosphoproteins: A‐kinase anchor protein 2 (AKAP2), chromogranin B (CHGB), and alpha‐parvin (PARVA), in IMAC‐enriched samples, whereas in TiO_2_‐enriched samples only myristoylated alanine‐rich C‐kinase substrate (MARCKS) strongly correlated with OS. Similarly, in the TN group, 13 proteins in TiO_2_‐enriched samples showed a strong positive correlation with OS, and none in the IMAC‐enriched samples. Interestingly, the majority of differentially expressed phosphoproteins correlated positively with OS, while only neurofilament medium polypeptide (NEFM; *r* = −0.42) and serine/arginine repetitive matrix protein 1 (SRRM1; *r* = −0.56) showed a moderate negative correlation (*r* = < −0.7 and > −0.3) with OS. Differentially expressed phosphoproteins that correlate with OS (with *r* > 0.4 or *r* < −0.4) in TN and NAT groups, both in IMAC‐ and TiO_2_‐enriched samples are listed in Table 3, along with information on their phospho‐site and roles in a variety of biological processes.

**Table 3:** List of differentially expressed phosphoproteins that correlate with overall survival. Information related to the biological processes was obtained from the web platform UniProt and GeneCards. Data is presented for phosphorylated proteins with correlation coefficient (*r*) value0s > 0.4 or < −0.4 in both the treatment‐naïve and neoadjuvantly treated group.

Phosphoprotein	*r* Value	Biological process	Phosphoprotein	*r* Value	Biological process
**IMAC: Treatment‐naïve**			AKAP12‐ S483	0.57	Cell growth, signal transduction
ERCC5‐S607	0.57	Excision/DNA repair	PARD3B‐S644	0.57	Cell cycle, cell division
MIA3‐S1745	0.56	Exocytosis, protein secretion	PPFIBP1‐S794	0.57	Focal adhesion
PKP4‐S337	0.49	Cytokinesis	MYLK‐T1778	0.56	Smooth muscle contraction
TGFB1I1‐S141	0.44	Differentiation, Wnt signaling	AHNAK‐S3426	0.55	Cell differentiation, migration
AHNAK‐S3426	0.43	Neuronal cell differentiation	FAM198B‐ S203	0.55	Unknown
ANKS1A‐S663	0.43	EGFR signaling	MEPCE‐S217	0.55	RNA binding
DENR‐T69	0.41	Protein biosynthesis	SRRM2‐T1413	0.55	mRNA processing, splicing
NEFM‐S633	−0.42	Maintenance of neuronal caliber	TNKS1BP1‐S691	0.52	Enzyme binding
**TiO**_**2**_ **: Treatment‐naive**			BICC1‐S971	0.51	Nucleic acid and RNA binding
IRF2BPL‐S547	0.94	Wnt‐signaling	EGFR‐S1121	0.51	Cell adhesion, growth, migration
PALLD‐S1118	0.92	Cytoskeleton organization	EPB41L1‐S819	0.51	Actin cytoskeleton organization
GIGYF2‐S221	0.82	Translation initiation	MARCKS‐S26	0.51	Cell motility, cell cycle
VCAN‐S2116	0.82	Cell adhesion, motility, growth	RBM17‐S62	0.51	mRNA processing, splicing
IMPDH2‐S91	0.79	Purine biosynthesis	SRSF6‐S316	0.51	mRNA processing, splicing
CHGA‐S300	0.78	Neuroendocrine peptide secretion	AMPD2‐S89	0.50	Nucleotide metabolism
KIF13B‐S1381	0.77	Cortical cytoskeleton organization	EPB41L1‐T550	0.50	Neuronal membrane plasticity
PALLD‐S1121	0.76	Cytoskeleton organization	SH3PXD2A‐S724	0.50	ECM degradation
PALLD‐S641	0.76	Cytoskeleton organization	TLN2‐T1843	0.50	Cell adhesion
SND1‐S426	0.74	Transcription regulation	AMPD2‐T88	0.49	Purine nucleotide metabolism
EPS15‐S796	0.72	Cell growth regulation	SRRM2‐S2123	0.49	mRNA processing, splicing
FLNA‐S2526	0.71	Cilium biogenesis/degradation	STX7‐S129	0.49	Protein trafficking
SEPTIN1‐S295	0.71	Cell cycle, cell division	ANKS1A‐S663	0.48	EGFR signaling
KIAA1217‐T1080	0.67	Skeletal system development	MARCKS‐S27	0.48	Cell motility, cell cycle
PPFIBP1‐S371	0.67	Focal adhesion	CLDN12‐S228	0.47	Cell‐adhesion
PTBP1‐S110	0.67	mRNA processing, splicing	IRF2BPL‐S69	0.47	Protein degradation
LTBP2‐S506	0.65	Elastic‐fiber architecture	PTRF‐S171	0.47	Transcription regulation
PDCD4‐S313	0.65	Apoptosis	SH3D19‐S430	0.47	Cytoskeletal organization
MARCKS‐S118	0.64	Cell motility, cell cycle	CCNY‐S324	0.46	Cell cycle regulation, Wnt signaling
STXBP5‐S85	0.62	Exocytosis, Vesicle transport	RSRC2‐S32	0.46	RNA binding
VCAN‐S1349	0.62	Cell adhesion, motility, growth	SPAG9‐T351	0.46	Cell cycle, cell division
DOCK2‐S1706	0.61	Cytoskeleton organization	VIM‐S459	0.46	Filament structure regulation
DNM1‐T849	0.60	Mitochondrial fission	AHNAK‐S5448	0.45	Neuronal cell differentiation
VCAN‐S362	0.60	Cell adhesion, motility, growth	DAP‐S3	0.45	Autophagy, cell death
VCAN‐S1351	0.56	Cell adhesion, motility, growth	MARCKS‐S145	0.45	Cell motility, cell cycle
AKAP12‐S1331	0.55	Cell growth, signal transduction	PHLDB2‐S334	0.45	Postsynaptic apparatus assembly
PDLIM3‐S93	0.53	Actin filament organization	CARTPT‐S48	0.44	Endogenous psychostimulant
VCAN‐S364	0.52	Cell adhesion, motility, growth	EIF3C‐S909	0.44	Protein biosynthesis
HDAC7‐S542	0.51	Transcription regulation	EPB41L3‐T693	0.44	Apoptosis, cell adhesion
NUMBL‐S283	0.46	Neurogenesis	NEFM‐S633	0.44	Maintenance of neuronal caliber
SRRM1‐T623	−0.56	mRNA processing, splicing	PDGFRA‐S527	0.44	Cell branching and adhesion
**IMAC: Neoadjuvantly treated**			ADD1‐S358	0.43	Actin cytoskeleton organization
AKAP2‐S748	0.74	Cell growth, migration	DOCK1‐S1779	0.43	Apoptosis, phagocytosis
CHGB‐S626	0.70	Secretory vesicle biogenesis	FLNA‐S1055	0.43	Cilium biogenesis/degradation
PARVA‐T16	0.70	Angiogenesis, cell adhesion	HBB‐S10	0.43	Oxygen transport
ARL3‐S5	0.69	Cell cycle, protein transport	NUCKS1‐S204	0.43	DNA damage, repair
DEPTOR‐S244	0.69	mTOR signaling	PPP1R12C‐S383	0.43	Signal transduction
GAB1‐S163	0.68	Cell growth, migration, apoptosis	MAP1B‐S1797	0.42	Neural development
AHNAK‐S511	0.65	Cell differentiation, migration	MAP1B‐T1932	0.42	Neural development
MECP2‐S423	0.64	Transcription regulation	NUCKS1‐S214	0.42	DNA damage, repair
SCG5‐T134	0.64	Intracellular protein transport	PAWR‐T230	0.42	Apoptosis, transcription regulation
ADD1‐S12	0.63	Actin cytoskeleton organization	RABGEF1‐S145	0.42	Endocytosis, protein transport
KIAA1671‐S385	0.63	Unknown	CFTR‐S707	0.41	Ion transport
PLEKHA6‐S468	0.63	Lipid biosynthesis	CHGA‐S333	0.41	Neuroendocrine peptide secretion
TERF2IP‐S32	0.63	Transcription regulation	IQSEC1‐S166	0.41	Protein transport
SCG5‐T137	0.60	Intracellular protein transport	RNF25‐S450	0.41	Ubl conjugation pathway
ADD1‐S353	0.59	Actin cytoskeleton organization	**TiO**_**2**_ **: Neoadjuvantly treated**		
AHNAK‐T4564	0.58	Cell differentiation, migration	MARCKS‐S118	0.81	Cell motility, cell cycle
HTATSF1‐S579	0.58	Transcription regulation	DNM1‐T849	0.45	Endocytosis, Necrosis
SRSF6‐S314	0.58	mRNA processing, splicing	AKAP12‐S627	0.44	Protein kinase A binding

### 3.10 Pancreatic cancer cells surviving the effects of chemotherapy express cancer stem cell‐like characteristics

**Fig. 6:**
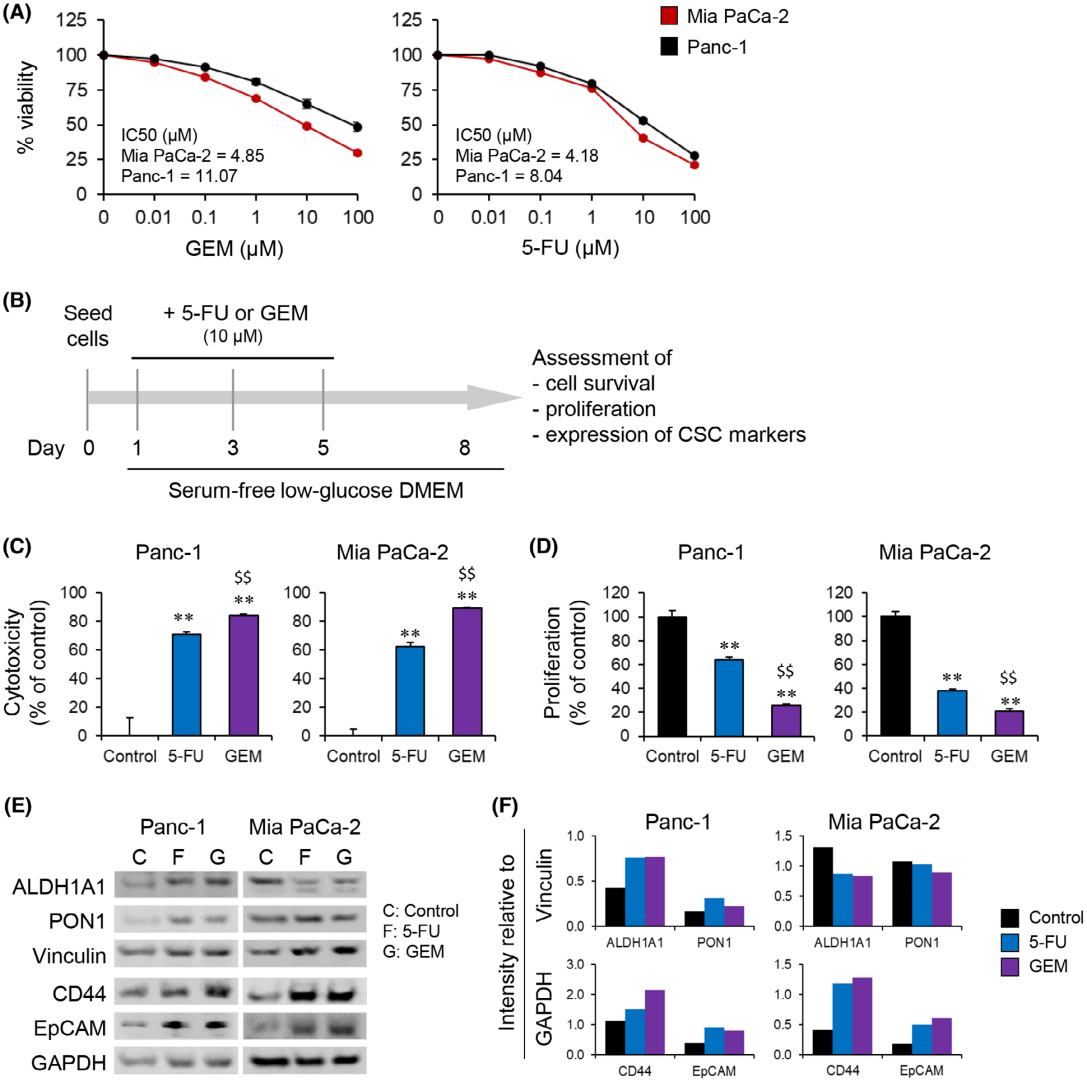
Assessment of chemoresistant cancer cells for stem cell‐like properties. (A) Dose response of cytotoxic effects of GEM and 5‐FU in Panc‐1 and Mia PaCa‐2 cells. (B–F) Panc‐1 and Mia PaCa‐2 cells pre‐treated with 10 μm 5‐FU or GEM, according to the experimental setup shown in B were investigated for drug‐induced cytotoxicity (C), proliferation (D), and expression of cancer stem cell markers (E, F). Seeding density of ~ 5000 and ~ 100 000 cells per well in a 96‐ or 6‐well plates was used for C–D and E–F, respectively. Drug‐induced cytotoxicity and proliferation were assessed using MTT assay and BrdU incorporation assay, respectively. (F) Protein expression quantification. Results are representative of three replicates. Error bars indicate SEM. Unpaired two‐tailed Student's *t*‐test with **P < 0.01, comparing 5‐FU or GEM with control, and ^$$^ *P* < 0.01 comparing 5‐FU and GEM. (E) Vinculin and GAPDH used as internal controls. 5‐FU, 5‐fluorouracil; GEM, gemcitabine.

A dose‐dependent reduction in cell viability was observed in both Panc‐1 and Mia PaCa‐2 cells following treatment with 5‐FU and GEM for 48 h (Fig. 6A). Next, cells were treated with either drug at a single concentration of 10 μm for repeated doses and assessed for the parameters indicated in the experimental timeline presented in Fig. 6B. Both drugs induced significant cell death in both cell lines compared with untreated control cells (Fig. 6C). However, the proportion of cells that survived the cytotoxic treatment differed between the cells lines: only 16% and 11% of Panc‐1 and Mia PaCa‐2 cells, respectively, survived GEM treatment, whereas the corresponding percentage were 29% and 38%, respectively, following 5‐FU exposure. A significant reduction in the proportion of actively proliferating cells was observed following exposure to either of the drugs: 36% and 62% reduction following 5‐FU treatment, and 74% and 79% reduction following GEM treatment of Panc‐1 and Mia PaCa‐2 cells, respectively (Fig. 6D). These findings suggest a more profound anti‐proliferative effect of GEM than 5‐FU in both cell lines, similar to the observed cytotoxic responses (Fig. 6C). Surviving cancer cells that managed to escape the cytotoxic effects of GEM or 5‐FU were investigated for the expression of four CSC markers: ALDH1A1, PON1, CD44, and EpCAM (Fig. 6E). Expression analysis revealed increased expression of all four CSC markers in Panc‐1 cells following exposure to 5‐FU or GEM treatment compared to control untreated cells (Fig. 6E,F). In contrast, Mia PaCa‐2 cells exhibited increased expression of CD44 and EpCAM in both 5‐FU and GEM treated cells compared with controls, whereas the expression of ALDH1A1 was moderately reduced and PON1 remained unaltered (Fig. 6E,F). Lastly, treatment‐induced change in the expression of ALDH1A1 was also investigated in HPAF‐II and BxPC‐3 cells. A significant increase in ALDH1A1 expression was observed in BxPC‐3 cells treated with both 5‐FU and GEM compared with control untreated cells; however, ALDH1A1 expression remained unchanged in HPAF‐II cells (Fig. S4A,B).

## 4 Discussion

In recent years, increasing evidence has demonstrated the benefits of NAT in PDAC treatment strategies, including reduction of the cancer burden, controlling metastasis, and ensuring a better selection of patients that may benefit from surgical resection [15]. Yet, the overall effects of treatment, especially in terms of survival, remain poor. Insufficient knowledge regarding the exact impact of NAT on tumors, including changes in tumor composition and cancer cell behavior, represents a major hurdle to further improvement of neoadjuvant treatment strategies. Currently, conventional radiological imaging and serum CA 19‐9 levels are used to assess the effect of NAT; however, these assessments do not accurately reflect treatment responses. Moreover, the unavailability of sufficient pretreatment tumor tissue that would enable direct comparison with surgically resected post‐NAT tissue and exact identification of treatment effects is a further limiting factor. To overcome these challenges, diverse approaches such as comparing residual tumors with adjacent non‐neoplastic tissue or pre‐NAT tumor‐derived organoids, blood biomarkers, and fine‐needle biopsies have been explored. However, progress has been limited, and further efforts are needed. To this end, we performed a comparative proteomic analysis to detect treatment‐induced molecular changes in tumors and to identify altered signaling pathways between NAT‐treated and TN tumors. The potential correlation between various clinical parameters, including OS and the proteins with differential expression and phosphorylation between TN and NAT tumors, were also investigated in this study.

**Fig. 7:**
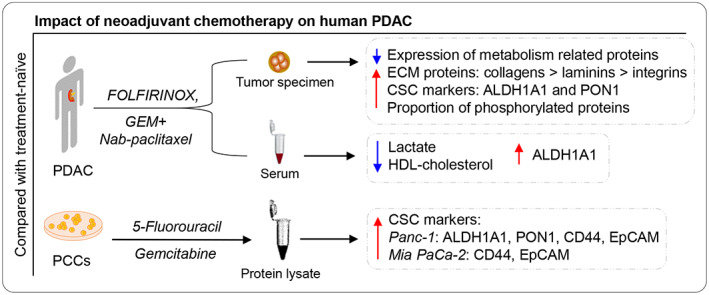
Impact of neoadjuvant chemotherapy on human PDAC. Compared with treatment‐naïve (TN) tumors, the neoadjuvantly treated (NAT) PDACs showed increased expression of metabolism‐related proteins, cancer stem cell (CSC) markers, and a proportion of phosphorylated proteins. Higher levels of the CSC marker ALDH1A1 and lower levels of lactate and HDL‐cholesterol were observed in matched serum samples from patients with NAT compared with patients with TN PDAC. In addition, pancreatic cancer cells (PCCs) that survived chemotherapy expressed high levels of the CSC markers ALDH1A1, PON1, CD44, and EpCAM as compared with control untreated cells.

A comparative analysis of the total‐ and phospho‐proteomic profiles of tumor tissues samples derived from NAT‐treated (*n* = 22) and TN (*n* = 20) PDACs was performed. Despite significant heterogeneity among the samples from each treatment group, 227 proteins that made up ~ 10% of all proteins selected for analysis showed altered expression in NAT tumors as compared to the TN tumors. Functionally, the total proteome detected covers a wide range of biological processes such as cell adhesion, metabolic regulation, ECM organization, and cell proliferation. Notably, ~ 78% of all DEPs showed lower expression in the NAT group than in TN group, suggesting a possible treatment‐induced down‐regulation of some biological processes in NAT tumors. Interestingly, the majority of DEPs (total 49 i.e., ~ 22%) are involved in the regulation of metabolic pathways, mainly glycolysis/gluconeogenesis, carbon metabolism, and amino acid metabolism, and 46 of these showed lower expression in NAT than in TN. Hence, the significantly lower expression of metabolism‐related proteins in NAT tumors indicates an alteration in tumor metabolic function following neoadjuvant chemotherapy (Fig. 7).

Following NAT, the residual tumor may typically show increased stromal content due to fibrosis in areas where, depending on the cytotoxic treatment effect, viable cancer cells are absent or reduced in number. Although no significant difference in the expression of stroma‐associated proteins was observed between NAT and TN samples, there was a trend toward higher expression of collagen and other ECM proteins in NAT than in TN samples. Consistent with this observation, histological assessment of H&E‐stained sections revealed collagen‐rich stroma with low cellularity in NAT compared to TN samples, which typically showed a cell‐rich tumor stroma with varying, more modest collagen content. Moreover, a lower proportion of various integrin, which are mainly expressed by cancer cells, was observed in NAT samples. These findings are in line with the anticipated treatment‐induced changes in PDAC following NAT. However, as histological assessment of tumor regression indicated moderate or poor response to NAT in the majority of cases (CAP‐2/3), changes in the expression of stoma‐related proteins are likely more pronounced in the small proportion of PDAC patients in whom NAT results in complete or near complete tumor regression (CAP‐0/1).

Surviving cancer cells in the residual tumor following NAT are chemoresistant and are considered to exhibit CSC‐like properties [42, 43, 44]. Accordingly, a significantly higher expression of the CSC markers ALDH1A1 and PON1 was seen in NAT compared to TN samples. Moreover, serum levels of ALDH1A1 were also higher in NAT patients than in the TN group, although serum PON1 levels were similar in both groups, and EpCAM levels were lower in NAT patients (Fig. 7). In several solid cancers, high expression and activity of ALDH1A1 are considered to be closely related to the stemness phenotype and to contribute to cancer progression, as well as being a biomarker for poor prognosis and low survival [45, 46, 47, 48]. Interestingly, the chemoresistant Panc‐1 and Mia PaCa‐2 cells that survived prolonged treatment with either 5‐FU or GEM also showed increased expression of CSC markers compared to untreated control cells, consistent with previous observations [49]. Increased expression of CSC markers differed between Panc‐1 and Mia PaCa‐2 cells, with increased levels of all four markers (ALDH1A1, PON1, CD44, and EpCAM) in Panc‐1 cells, whereas only CD44 and EpCAM were elevated in Mia PaCa‐2 cells. The observed difference in expression patterns could be partially explained by phenotypic and genotypic differences between the two cell lines [50].

As expected, post‐surgical OS was significantly lower in patients in the NAT group than those in TN group, as 73% of patients in the NAT group had BR/LA disease, and this proportion was merely 10% in the TN group. Interestingly, most of the metabolic genes with differential expression in NAT versus TN tumors correlated with OS in the NAT group. Notably, glycolysis pathway‐associated proteins showed a negative correlation with OS. Recent evidence suggests that glycolysis promotes disease progression, increases cancer stemness, and reduces chemosensitivity in PDAC [51, 52, 53].

Two different enrichment methods, IMAC and TiO_2_, were employed to obtain an as complete picture of the phosphorylation events. Both methods offer comparable enrichment efficiency; however, phosphopeptides that are unique to each method differ owing to the enrichment of different motifs [54]. The majority of phosphoproteins detected were common to both enrichment methods; however, unique proteins with differential phosphorylation between NAT and TN samples differed significantly between IMAC (*n* = 122) and TiO_2_ (*n* = 31). Majority of phospho‐events were associated with higher phosphorylation following NAT compared to TN. The majority of the phosphorylated proteins detected are known to be involved in the cytoskeletal organization or belong to cellular components and organelles. As the differentially expressed phosphoproteins mainly belong to the categories of cytoskeleton remodeling and organization as well as cell motility and migration, they may influence tumor cell migration and metastatic potential [55, 56]. Notably, of all differentially expressed phosphoproteins, only eight have a known association with metabolic processes and none of these correlate with OS.

The substantial differences observed in the proteomics data related to protein expression of metabolism‐associated genes in both treatment groups prompted us to investigate whether these alterations were limited to local events in the tumor or may have an impact at the systemic level. While there were no differences in BMI or serum albumin, total cholesterol and LDL‐cholesterol levels between the NAT and TN group, the serum levels of lactate and HDL‐cholesterol were significantly lower in NAT than in TN group. The latter differences may indicate some alterations in systemic metabolic function, possibly due to the effects of NAT treatment. Altered serum lipid levels, particularly reduced total and HDL cholesterol following radiotherapy, have been reported in breast cancer patients [57, 58]. Moreover, a recent unpublished study showed reduced plasma HDL‐cholesterol in PDAC patients compared with healthy controls [59]. Interestingly, the reduced serum lactate levels in NAT compared with TN patients are consistent with the observed reduced expression of tumor glycometabolic markers in the proteomics data. However, further investigations into the effects of NAT treatment on the levels of systemic metabolic markers are required to understand the nature and consequences of metabolic changes during NAT, particularly their potential clinical relevance.

This study has certain limitations. First, the NAT group includes a higher proportion of patients with advanced disease stages, which may have impact on the molecular characteristics of the tumors. Neoadjuvant chemotherapy may affect tumor metabolism, which might have contributed to the observed reduced metabolism in NAT samples compared with TN. Second, the comparison between NAT and TN samples is indirect since the actual impact of NAT in a given tumor cannot be assessed owing to the lack of a corresponding pre‐treatment tissue sample for comparative analysis. Lastly, because a random tissue sample was collected from each tumor, the presence of prominent heterogeneity in PDAC could influence the results.

## 5 Conclusions

To the best of our knowledge, this is the first study to report preliminary evidence of the effects of NAT on PDAC metabolism. Tumors treated with neoadjuvant chemotherapy showed significantly lower expression of metabolic proteins, particularly glycometabolic pathway markers. Reduced serum levels of lactate and cholesterol in patients who underwent NAT indicate that NAT treatment also induces systemic metabolic changes. Lastly, cancer cells that survived cytotoxic treatment were found to express increased levels of CSC markers, both *in vivo* and *in vitro*.

## Conflict of interest

The authors declare no conflict of interest.

## Author contributions

MA involved in conceptualization, data curation, investigation, methodology, project administration, writing—original draft, writing–review, and editing. CSV involved in data curation, investigation, methodology, funding acquisition, writing, review, and editing. AVF and LD involved in data curation and methodology. KJL involved in data curation, resources, writing, review, and editing. IPG involved in conceptualization, resources, supervision, funding acquisition, project administration, writing, review, and editing.

## Supporting information


**Fig. S1.** Expression pattern of differentially expressed proteins in TN versus NAT group.
**Fig. S2.** Correlation between protein expression and survival.
**Fig. S3.** Distribution of differentially expressed phosphoproteins.
**Fig. S4.** ALDH1A1 expression in BxPC‐3 and HPAF‐II cells.

**Table S1.** Clinicopathological information on the study series.
**Table S2.** MS data and enrichment analysis of total proteome.
**Table S3.** MS data and pathway analysis of DEPs between TN and NAT group.
**Table S4.** MS data for phospho‐proteome.
**Table S5.** MS data on differentially expressed phosphoproteins in TN versus NAT group.

## Data Availability

The datasets analyzed in this study are available in the supplementary data files. Additional information is available on request to the corresponding author.
