# Supplementary material for: Neoadjuvant chemotherapy is associated with an altered metabolic profile and increased cancer stemness in patients with pancreatic ductal adenocarcinoma
Source: Mol Oncol. 2022 Dec 5;17(1):59–81. doi: 10.1002/1878-0261.13344 (PMC9812839; doi:10.1002/1878-0261.13344)
Supplement: Supplementary file 1 — Fig. S1. Expression pattern of differentially expressed proteins in TN versus NAT group. Fig. S2. Correlation between protein expression and survival. Fig. S3. Distribution of differentially expressed phosphoproteins. Fig. S4. ALDH1A1 expression in BxPC‐3 and HPAF‐II cells. [file MOL2-17-59-s001.pdf]

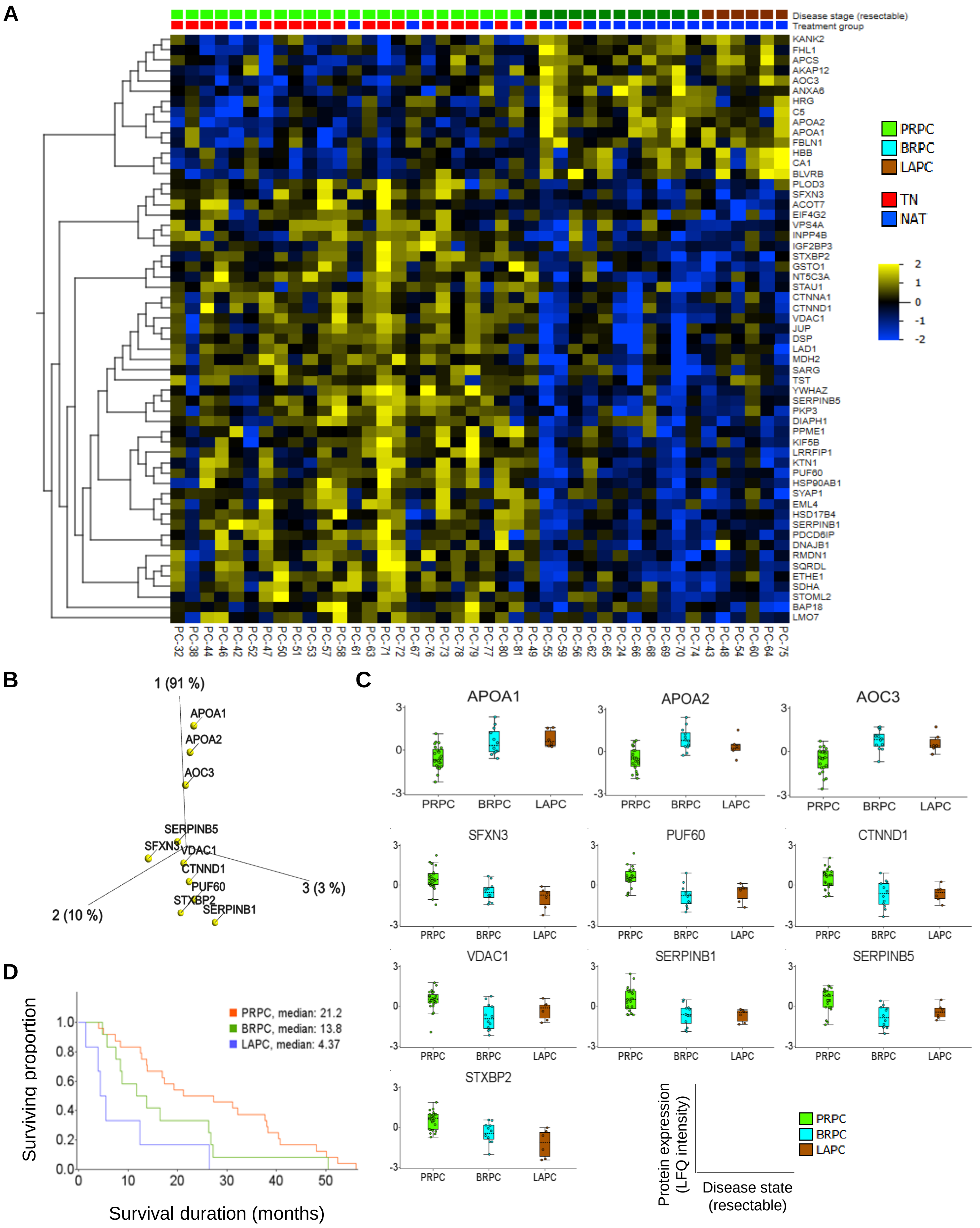

Fig. S1. A. Heat map of differentially expressed proteins between treatment naïve (TN) and neoadjuvantly treated (NAT) tumors when compared based on disease state irrespective to treatment group ($p<0.01$). **B.** PCA-plot of ten most differentially expressed protein (selected from **A**). **C.** Box plots showing expression pattern of proteins (shown in B). **D.** Kaplan-Meier survival estimation based on disease state, irrespective of treatment group. Survival duration was calculated from the date of surgery. PC, pancreatic cancer; PR, primary resectable PC; BR, borderline resectable PC; LA, locally advanced PC.

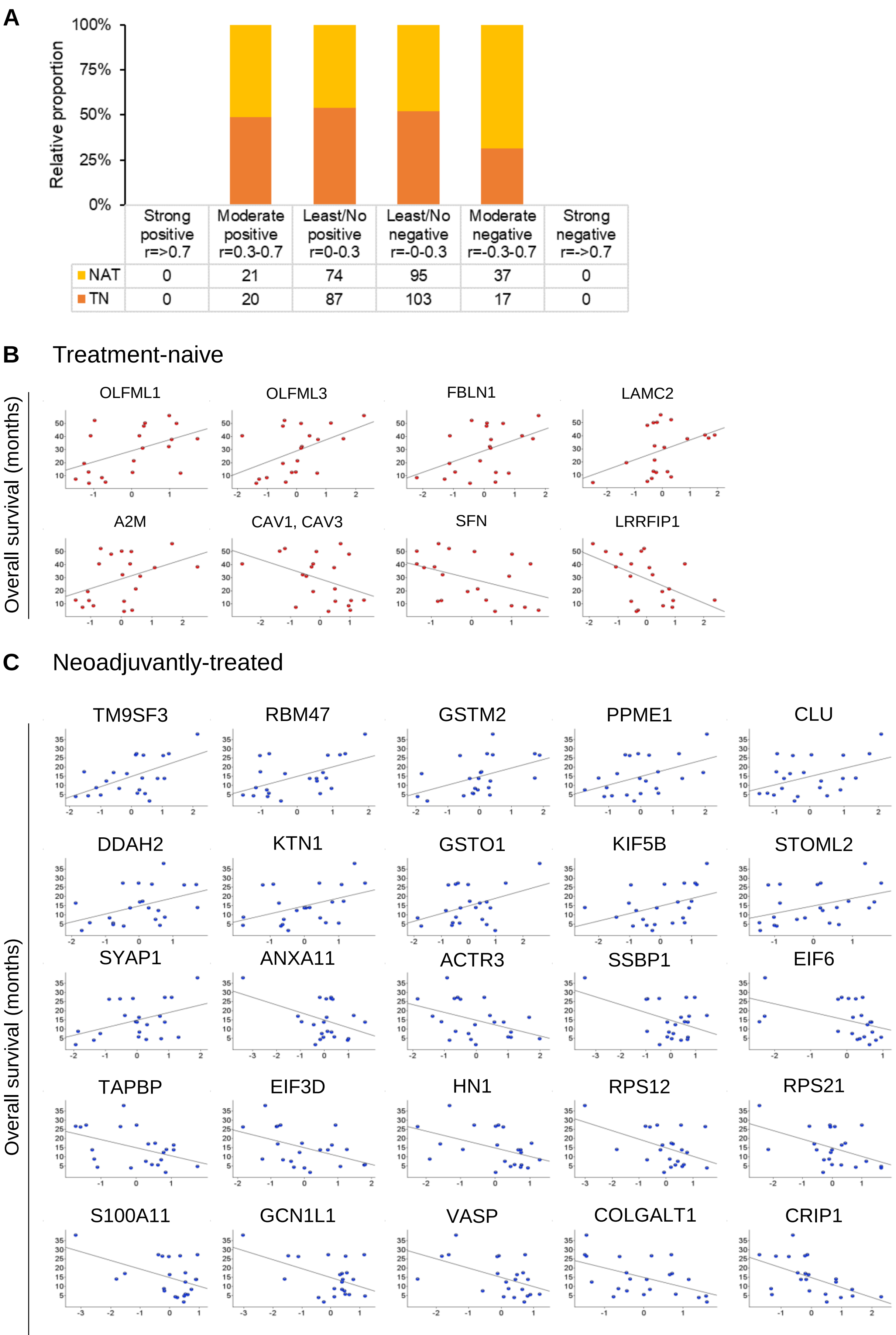

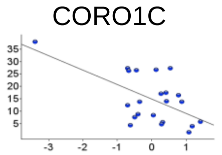

Fig. S2. A. Overview of the number of total proteins that correlate with overall survival. **B-C.** Scatterplots showing correlation between expression of differentially expressed proteins and overall survival for **B.** treatment naïve, and **C.** for neoadjuvantly-treated samples.

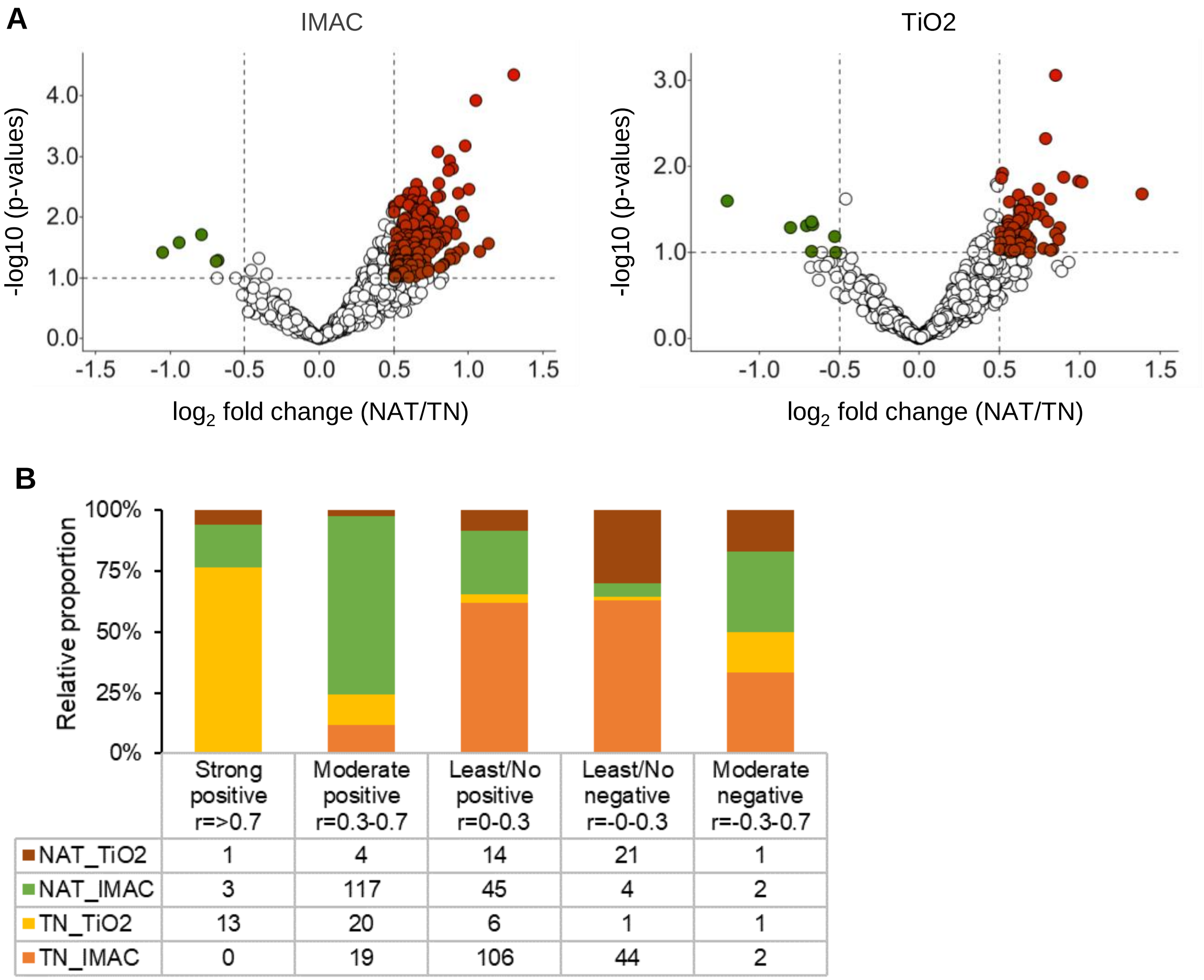

	Strong positive r=>0.7	Moderate positive r=0.3-0.7	Least/No positive r=0-0.3	Least/No negative r=-0-0.3	Moderate negative r=-0.3-0.7
NAT_TiO2	1	4	14	21	1
NAT_IMAC	3	117	45	4	2
TN_TiO2	13	20	6	1	1
TN_IMAC	0	19	106	44	2

Fig. S3. A. Volcano plots for differentially expressed phosphoproteins between NAT and TN for both IMAC- and TiO2-enriched samples. **B.** Overview of the number of phosphoproteins that correlate with overall survival. IMAC immobilized metal affinity chromatography; TiO2, titanium dioxide; NAT, neoadjuvantly-treated; TN, treatment naïve; r, correlation coefficient.

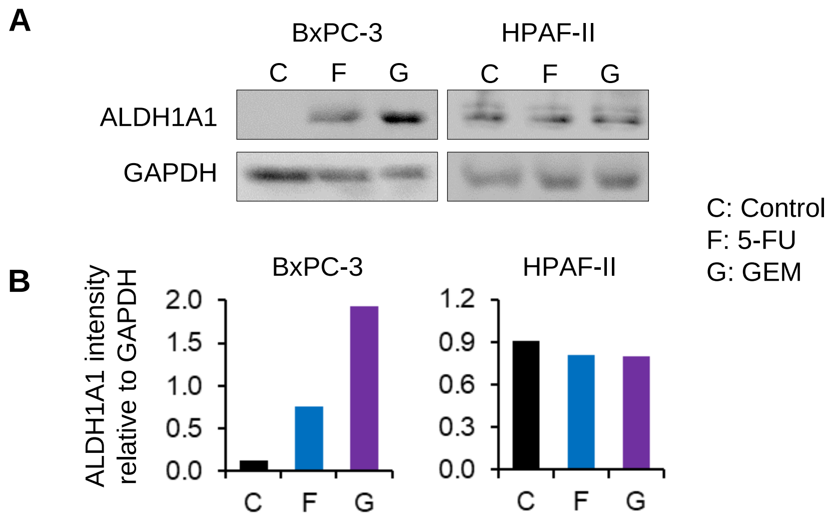

Fig. S4. A. Representative western blot images showing ALDH1A1 expression in BxPC-3 and HPAF-II cells treated with 10 µM 5-FU or GEM, according to the experimental setup shown in Fig. 6B. GAPDH used as internal control. **B.** Quantification of ALDH1A1 expression intensity. ALDH1A1, aldehyde dehydrogenase 1; 5-FU, 5-fluorouracil; GEM, gemcitabine.
